# Pulmonary Epithelium

**Published:** 1857-12

**Authors:** 


*Pulmonary Epithelium.*—In the July number of the British and Foreign Medico-Chirurgical Review, we notice an article by Dr. C. Radclyffe Hall, on the epithelium of the air vesicles of the human lung.

Some of our readers may recollect a paper by Dr. Hall in the last October number of the Review, in which he states that in chronic pulmonary consumption, "fatty atrophy of the epithelium of the air vesicles is antecedent to the formation of tubercle; in the same number we have a paper by Mr. Rainey, against the existence of epithelium in the air vesicles. This paper by Dr. Hall assumes to demonstrate these epithelial cells, and is illustrated by wood cuts of their microscopic appearance as seen by Dr. Hall and Dr. Brittan. Assuming that these illustrations are correct, and we have no reason to doubt it, there seems no doubt but that epithelial cells can be demonstrated in the human lungs, and the lungs of the mammalia. We cannot but agree with Dr. Hall, that on a question of relative authority, "the positive evidences of one trustworthy observer is usually allowed to over-rule the negative evidence of many." An examination of this article by Dr. Hall, with the drawing of the microscopic appearances by Dr. Brittan, will not fail to convince any one of the existence of epithelium in this situation. If this were not the fact, the statement of Dr. Hall that fatty degeneration of the epithelium of the air cells preceded the deposition of tubercle, (an observation which we conceive to be of value, and which we hope may be further elaborated) would of course be entirely without foundation.

